# A Low-Viscosity BisGMA Derivative for Resin Composites: Synthesis, Characterization, and Evaluation of Its Rheological Properties

**DOI:** 10.3390/ma14020338

**Published:** 2021-01-11

**Authors:** Ali Alrahlah, Abdel-Basit Al-Odayni, Haifa Fahad Al-Mutairi, Bashaer Mousa Almousa, Faisal S. Alsubaie, Rawaiz Khan, Waseem Sharaf Saeed

**Affiliations:** 1 Restorative Dental Sciences Department, College of Dentistry, King Saud University, Riyadh 11545, Saudi Arabia; 2 Engineer Abdullah Bugshan Research Chair for Dental and Oral Rehabilitation, College of Dentistry, King Saud University, Riyadh 11545, Saudi Arabia; haifafmutairi@gmail.com (H.F.A.-M.); bashaermousa@gmail.com (B.M.A.); krawaiz@ksu.edu.sa (R.K.); wsaeed@ksu.edu.sa (W.S.S.); 3 Chemistry Department, College of Science, King Saud University, P.O. Box 2455, Riyadh 11451, Saudi Arabia; 437102504@student.ksu.edu.sa

**Keywords:** BisGMA derivative, dental resin composite, monomer viscosity, dental material, rheometry

## Abstract

This study aimed to synthesize new bisphenol A-glycidyl methacrylate (BisGMA) derivatives, targeting a reduction in its viscosity by substituting one of its OH groups, the leading cause of its high viscosity, with a chlorine atom. Hence, this monochloro-BisGMA (mCl-BisGMA) monomer was synthesized by Appel reaction procedure, and its structure was confirmed using Fourier transform infrared spectroscopy, ^1^H and ^13^C-nuclear magnetic resonance spectroscopy, and mass spectroscopy. The viscosity of mCl-BisGMA (8.3 Pa·s) was measured under rheometry conditions, and it was found to be more than 65-fold lower than that of BisGMA (566.1 Pa·s) at 25 °C. For the assessment of the viscosity changes of model resins in the presence of mCl-BisGMA, a series of resin matrices, in which, besides BisGMA, 50 wt % was triethylene glycol dimethacrylate, were prepared and evaluated at 20, 25, and 35 °C. Thus, BisGMA was incrementally replaced by 25% mCl-BisGMA to obtain TBC0, TBC25, TBC50, TBC75, and TBC100 blends. The viscosity decreased with temperature, and the mCl-BisGMA content in the resin mixture increased. The substantial reduction in the viscosity value of mCl-BisGMA compared with that of BisGMA may imply its potential use as a dental resin matrix, either alone or in combination with traditional monomers. However, the various properties of mCl-BisGMA-containing matrices should be evaluated.

## 1. Introduction

Dental caries is one of the most common chronic diseases throughout the life span. Although it is largely preventable, it is still a major public health problem worldwide [1]. Restorative dental materials used to fill dental cavities are usually metals, ceramics, polymers, or composites [2]. Photocurable resin-based composites are the common choice for patients and practitioners due to their superior aesthetics and easy handling and shaping properties [3]. Therefore, they increasingly became popular in dentistry. Generally, resin-based composites comprise resin matrix, reinforcing fillers, filler–matrix interface coupling agents, and polymerization initiator systems; however, the last two are usually incorporated in traces (<1 wt %). Resins are conventionally photocurable acrylate-based monomers, which represent one major influencer of the final properties of filling composites. Matrices of commercial dental composites predominantly consist of bisphenol A-glycidyl methacrylate (BisGMA) monomer [4,5,6]. However, other dimethacrylates, such as urethane dimethacrylate (UDMA), ethoxylated bisphenol A dimethacrylate (BisEMA), and triethylene glycol dimethacrylate (TEGDMA), are also common constituents of the resin matrix [7,8].

BisGMA is a good choice as a base matrix for hosting composite constituents. It has strong advantages over its (di)(meth)acrylate-based analogs, including its low polymerization shrinkage, suitable mechanical properties, relatively high refractive index, and excellent adhesion to enamel [9,10]. The major disadvantage of BisGMA is its high viscosity [9], which prevents the addition of high contents of fillers. On the molecular level, BisGMA has a stiff central aromatic core and two hydroxyl groups that kinetically cause a reduced degree of molecular freedom. Hydrogen bonds are said to be the major player in the degree of intermolecular interaction; thus, they increase the viscosity of the monomer, reduce its chains’ mobility, and make it difficult to mix with other ingredients, resulting in a reduced degree of conversion (DC) upon polymerization. These impair the overall integrity and longevity of the restorative materials; in particular, the mechanical properties are significantly weakened.

The viscosity of the matrix is of great importance and is strictly associated with certain features of the composite, either before or after application [11]. The high viscosity of the matrix may limit the amounts of reinforcing materials, generate handling difficulties, reduce DC, weaken composite mechanical properties, and reduce its longevity. It is directly linked to the chemical structure of monomeric constituents; thus, it is an indicator of the degree of intermolecular interactions at the molecular level. However, the viscosity of BisGMA (910 Pa·s, at ~22 °C) [12] is the highest among the other common dimethacrylate monomers used in resin-based dental composite due to its large molecular weight and strong molecular interactions driven by H-bonding.

To overcome the drawbacks correlated with the high viscosity of BisGMA, the matrix is usually diluted with TEGDMA (0.01 Pa·s, at ~22 °C) as a suitable low-viscosity diluent [12]. However, TEGDMA enhances the hydrophilicity characters of the composite, which further increases the undesirable water sorption and polymerization shrinkage. Additionally, (di)(multi)functional monomers form cross-linked networks, which causes a remarkable decrease in DC upon curing. As polymerization proceeds, the movement of the macro-radicals becomes more restricted, and thereafter, diffusion-controlled process being the dominant mechanism of propagation and termination [6,13]. To obtain suitable low-viscose monomers, scientists have synthesized different BisGMA-alike monomers [7,9]. Others tried the fabrication of the BisGMA chemical structure by, for example, synthesizing various derivatives [5,12,14,15,16,17]; however, reaching the state-of-the-art monomer with low viscosity and superior properties for dental restorations is one current objective for specialists.

In this regard, the replacement of the hydroxyl groups in BisGMA by, e.g., less-hydrophilic groups, is one possible solution to tailor its viscosity, i.e., by limiting the degree of molecular interaction caused by H-bonding. Examples of these organic groups include –CH_3_ [16], –CF_3_ [17], –OCH_3_ [15], –OCOCH_2_C_6_H_5_ and –C_4_H_9_ [14], and –Cl [12]. In the latter case, Al-Odayni et al. [12] synthesized a new BisGMA derivative (Cl-BisGMA, hereafter termed as dCl-BisGMA) in which the two hydroxyls were substituted by chlorine atoms. Remarkable enhancements in the viscosity, DC, and water sorption were reported after modification. However, of the studied properties, water solubility was increased. Additionally, the initial biological evaluation of dCl-BisGMA, assessed through cell viability and live/dead assays and cell attachment tests using immortalized human bone marrow stromal cells, revealed biocompatible characters, encouraging further investigations. Further, targeting only one hydroxyl group from the two in BisGMA may sufficiently decrease its viscosity and additionally, retain its desirable properties.

The objective of this work is to develop a new BisGMA derivative in which one hydroxyl is replaced by a chlorine atom. It is expected that the replacement of one hydroxyl group would significantly reduce viscosity. Compared with BisGMA and dCl-BisGMA, monochloro-BisGMA (mCl-BisGMA) should possess greater advantages. It was synthesized using chemically controlled reaction conditions; one –OH group was targeted for substitution, and afterward, its structure was fully characterized using Fourier transform infrared (FTIR), ^1^H and ^13^C-nuclear magnetic resonance (NMR) spectroscopy, and mass spectroscopy (MS). The viscosities of mCl-BisGMA alone and in a mixture with BisGMA and TEGDMA were also evaluated at different temperatures.

## 2. Materials and Methods

### 2.1. Materials

BisGMA (>98%), TEGDMA (>95%), 2-(N, N-dimethyl amino) ethyl-methacrylate (98%), camphorquinone (97%), and carbon tetrachloride (CCl_4_; >99.8%) were procured from Sigma-Aldrich, Darmstadt, Germany. Triphenylphosphine (Ph_3_P; >98%) was purchased from Cica-reagent (Kanto Chemical, Tokyo, Japan). n-Hexane (n-H; >97%) was obtained from Avonchem, Cheshire, UK. Ethyl acetate (EA; 99%) and dichloromethane (DCM; 99%) were obtained from Fisher Scientific, Leicestershire, UK. All chemicals were used as received, without further purification unless otherwise stated.

### 2.2. Methods

#### 2.2.1. Synthesis of mCl-BisGMA Monomer

The targeted mCl-BisGMA monomer was synthesized using the well-known Appel reaction procedure [18,19,20] with a slight modification of the protocol as described earlier [12]. BisGMA (0.039 mol, 20 g) was completely dissolved in 65 mL of DCM and stirred for 30 min, while a low stream of nitrogen gas was passed through the system for deoxygenation, i.e., to provide an inert reaction environment. An equimolar amount of 0.039 mol (3.8 mL) of CCl_4_ was added to the solution and stirred for 10 min, and subsequently, 17.77 g of the catalyst, Ph_3_P, was added. After the complete dissolution of the catalyst, the reaction mixture was stirred under reflux for 3 h and further for 6 h at room temperature (24 °C ± 2 °C). The quantity of the solvent was reduced under vacuum using a rotary evaporator at 30 °C; afterward, the catalyst by-product, triphenylphosphine oxide (Ph_3_P-O), was precipitated using a 1:1 solvent mixture of EA/*n*-H (*v*/*v*) and left to settle overnight. The supernatant was collected (by decantation process), and the solvent was removed in the rotary evaporator. The crude product was purified through a flash column chromatography (FCC) using silica gel (60 mesh) as a stationary phase and an EA/*n*-H elution system (4:6 by volume; mobile phase) [21]. Fractions collected from FCC indicated the presence of two dominant components: one was previously identified as dCl-BisGMA [12], and the other with relatively high polarity was further identified as the mCl-BisGMA (thin-layer chromatography (TLC, 0.1 mm thick silica gel coated aluminum plate) retention factor, RF = 0.81 and 0.47 of di- and mono-Cl-BisGMA, respectively). After the removal of the solvents, 6 g (29%) of a light-yellow product, the final isolated yield, was obtained. Further, an adequate amount of the product was prepared for the next experiments and kept in a dark container at 8 °C until used.

#### 2.2.2. Preparation of Resins

The resins were formulated as shown in Table 1. The BisGMA was replaced as 0%, 25%, 50%, 75%, and 100% by mass of the new monomer, mCl-BisGMA, coded as TBC0–TBC100. As seen in Table 1, in each case, 50% of the resin mixture constitutes TEGDMA, the traditional diluent monomer.

#### 2.2.3. Characterization

The chemical structure of the synthesized mCl-BisGMA monomer was confirmed by various techniques, including FTIR spectroscopy, NMR spectroscopy, and MS. FTIR spectra were recorded from KBr pellets using a Nicolet iS10 spectrometer (Thermo Scientific, Madison, WI, USA) over the range of 4000–400 cm^−1^, resolution of 4 cm^−1^, and scanning cycles of 32 per spectrum. The NMR spectra were obtained using a DELTA NMR spectrometer (JEOL Resonance, JEOL, Tokyo, Japan) operating at 400 MHz, using deuterated chloroform (CDCl_3_) as the solvent and tetramethylsilane as the internal standard. The data were visualized using the Delta v5.0.4 software (JEOL, Tokyo, Japan), and the chemical shift was presented in parts per million (ppm). The mass spectrum was registered using an Accu-TOF LC-plus JMS-T100 LP ToF-MS spectrometer (JEOL, Tokyo, Japan) equipped with a direct analysis in real-time (DART) ion source (IonSense, Saugus, MA, USA) and operated in the positive-ion mode. Fragments were obtained at 350 °C with a peak voltage of 500 V; the machine was calibrated using PEG200 for 0.99–1.16 min. Selected peaks were assigned using the MassCentre software (version 1.3.m, JEOL, Tokyo, Japan).

### 2.3. Rheological Studies

The rheological properties of the monomers, including the newly synthesized mCl-BisGMA monomers, and the resin mixtures (TBC0–TBC100) were measured using MCR-72 rheometer (Anton Paar, Graz, Austria) in the rotational mode under a steady shear sweep over the range of 0.01–1000 1/s using a 25-parallel plate geometry and 0.25-mm gap at different measuring temperatures (20, 25, and 35 °C). Due to the large variations in the viscosity values of the materials under investigation and the limitation of the used rheometer torque, each sample was recorded only in the corresponding linear viscosity values, and at least four replication tests (n = 4) were performed for each sample.

## 3. Results and Discussion

### 3.1. Characterization of mCl-BisGMA

**Figure 1:**
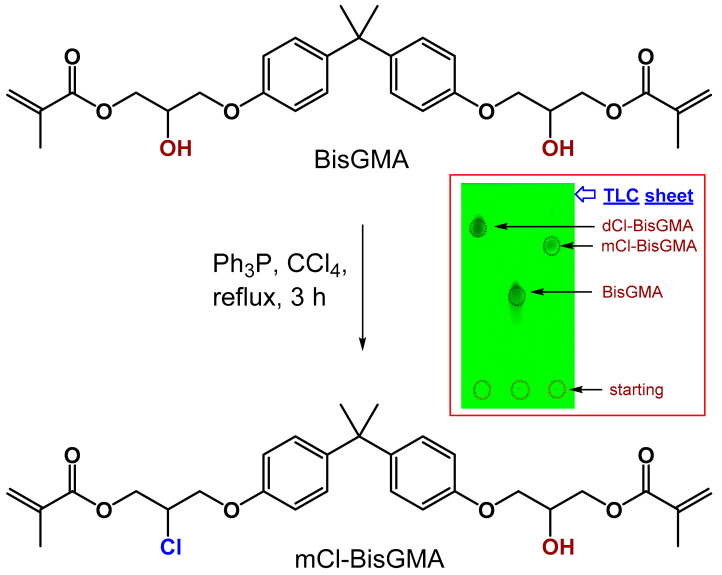
Synthesis of mCl-BisGMA monomer. Inset shows the mobility of dCl-BisGMA, mCl-BisGMA, and BisGMA monomers’ TLC spots using EA/n-H (4:6 *v*/*v*) as a mobile phase. Spots were visualized under UV light.

The chemical structure of mCl-BisGMA was confirmed by FTIR, ^1^H and ^13^C-NMR, and MS. Figure 1 shows the schematic illustration of the chemical reaction in which one hydroxyl group was replaced by a chlorine atom. The experiment was designed to be controlled by the amount of chlorine source (CCl_4_); thus, an amount (by mole) of CCl_4_ equal to only one mole of the hydroxyl group in BisGMA was used. However, the substitution of either one or both OH groups on BisGMA was expected, and it was confirmed by TLC in which three dominant spots were observed at RFs of 0.81, 0.47, and 0.21. These spots were identified, respectively, for dCl-BisGMA, mCl-BisGMA, and BisGMA, as shown in Figure 1 (inset). The spot with the highest RF (0.81) was previously confirmed for dCl-BisGMA [12]; therefore, the second with a relatively low RF value of 0.47 was assigned for the mCl-BisGMA. Generally, RF is proportional to the material hydrophilicity character, solvent type, and stationary phase properties. Here as the mobile phase, EA/n-H (4:6 by volume), is more hydrophobic, dCl-BisGMA is more likely to move relatively fast followed by mCl-BisGMA compared with BisGMA (the highest hydrophilic component in the mixture). After the separation of the two spots using column chromatography, the isolated materials were subjected to fully analytical characterization as detailed below. The reaction mechanism generally occurred through a second-order nucleophilic reaction (S_N_2); therefore, an inverted configuration was expected [12]. The dCl-BisGMA yield was higher than that of mCl-BisGMA at approximately 50% and 29%, respectively. This may suggest that the new geometry of mCl-BisGMA makes it more reactive than BisGMA, facilitating the action of the catalyst, and thus, the conversion of mCl-BisGMA into dCl-BisGMA is facilitated. However, more evidence is required for this argument; thus, verification may be one future task.

BisGMA = FTIR [ν; cm^−1^]: 3419 (–OH), 1714 (C=O), 1636 (C=C aliphatic), 1607 (C=C aromatic); ^1^H-NMR (400 MHz, CDCl_3_) [δ; ppm]: 7.42–7.10 (m, 4H, H-10), 7.04–6.79 (m, 4H, H-9), 6.22 (d, J = 12.5 Hz, 2H, H-2a), 5.68 (d, J = 12.5 Hz, 2H, H-2b), 4.57–3.70 (m, 10H, H-6, H-5 and H-7), 3.18 (s, 2H, H-14), 2.04 (s, 6H, H-1), 1.72 (s, 6H, H-13); ^13^C-NMR: 167.02 (C-4), 155.85 (C-8), 143.09 (C-11), 135.46 (C-3), 127.29 (C-10), 125.92 (C-2), 113.54 (C-9), 77.00 (CDCl_3_), 68.38 (C-7), 67.82 (C-6), 65.22 (C-5), 41.19 (C-12), 30.58 (C-13), 17.82 (C-1). mCl-BisGMA = clear, light-yellow liquid; 29% yield; Mass [m/z; mu]: 530.21503 (C_29_H_35_^35^ClO_7_) [M]^+.^, 531.22282 (C_29_H_36_^35^ClO_7_) [M+H]^+^, 533.22513 (C_29_H_36_^37^ClO_7_) [(M+H)+2]^+^; FTIR [ν; cm^−1^]: 3487 (–OH), 1720 (C=O), 1637 (C=C aliphatic), 1608 (C=C aromatic); ^1^H-NMR (400 MHz, CDCl_3_) [δ; ppm]: 7.40–7.10 (m, 4H, H-10), 7.02–6.78 (m, 4H, H-9), 6.34–6.13 (m, 2H, H-2a), 5.79–5.58 (m, 2H, H-2b), 4.73–3.69 (m, 10H, H-7, H-6, and H-5), 2.99 (s, 1H, H-14), 2.04 (s, 6H, H-1), 1.72 (s, 6H, H-13); ^13^C-NMR: 167.29 (C-4), 166.59 (C-8), 143.69 (C-11), 135.66 (C-3), 127.70 (C-10), 126.16 (C-2), 114.34–113.45 (C-9), 77.00 (CDCl_3_), 68.51 (C-7), 65.49 (C-5), 64.66 (C-6), 41.60 (C-12), 31.21–30.43 (C-13), 18.12 (C-1).

#### 3.1.1. FTIR Analysis

**Figure 2:**
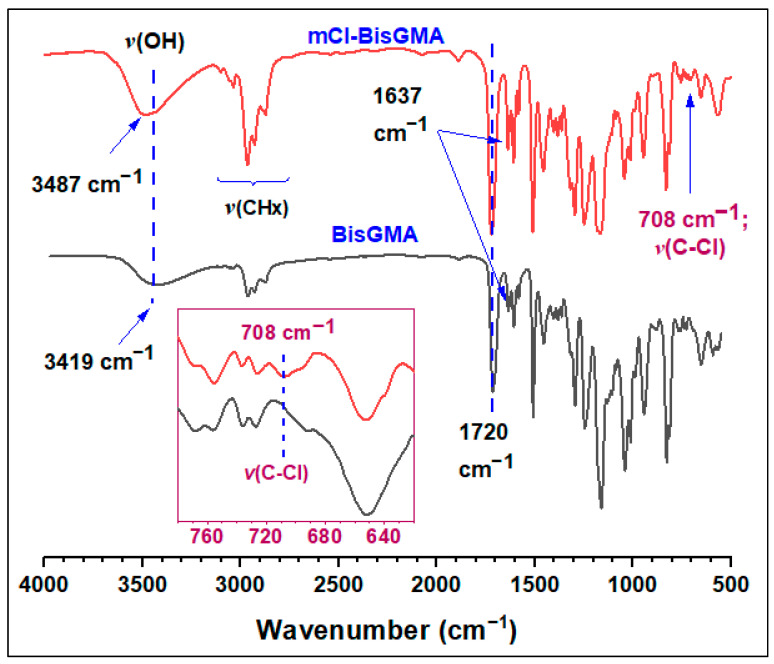
FTIR spectra of BisGMA and mCl-BisGMA monomers. The insert is a magnification of the C–Cl bond stretching vibrational region.

Figure 2 shows the FTIR spectra of mCl-BisGMA and BisGMA monomers. The BisGMA spectrum is similar to the one reported in the literature [5,12] in which the broadband at 3416 cm^−1^ is a characteristic of –OH stretching vibration. The peaks of C=O, C=C acrylic, and C=C aromatic were evident at 1714, 1636, and 1607 cm^−1^, respectively. However, because of the presence of chlorine atom in mCl-BisGMA structure, a slight shift in the whole spectrum toward relatively high frequencies was observed; thus, –OH, C=O, C=C vinylic, and C=C aromatic appeared at 3487, 1720, 1637, and 1608 cm^−1^, respectively. This may indicate a reduction in the degree of hydrogen bonding due to the partial replacement of –OH by Cl. In the spectra of mCl-BisGMA, the peak corresponding to the new C–Cl bond was observed at 708 cm^−1^ [12,22], confirming the success of the intended substitution.

#### 3.1.2. NMR Analysis

**Figure 3:**
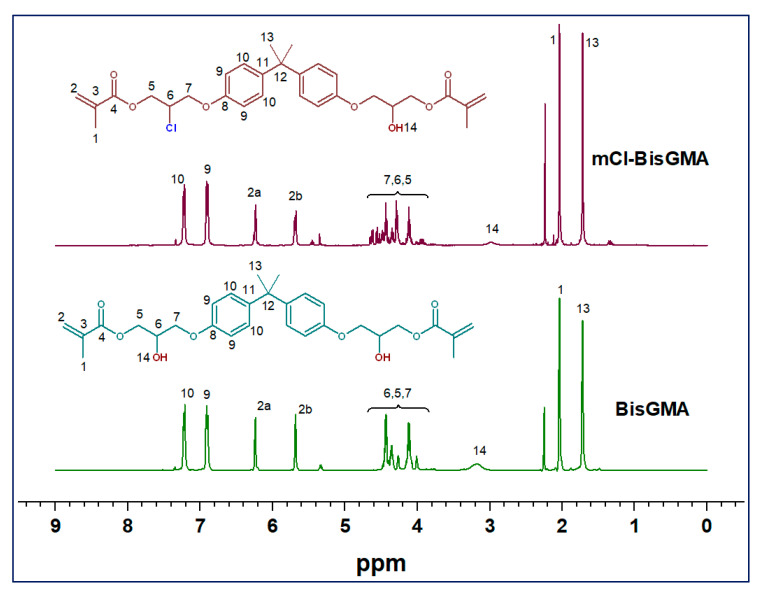
^1^H-NMR spectra of BisGMA and mCl-BisGMA in CDCl_3_.

**Figure 4:**
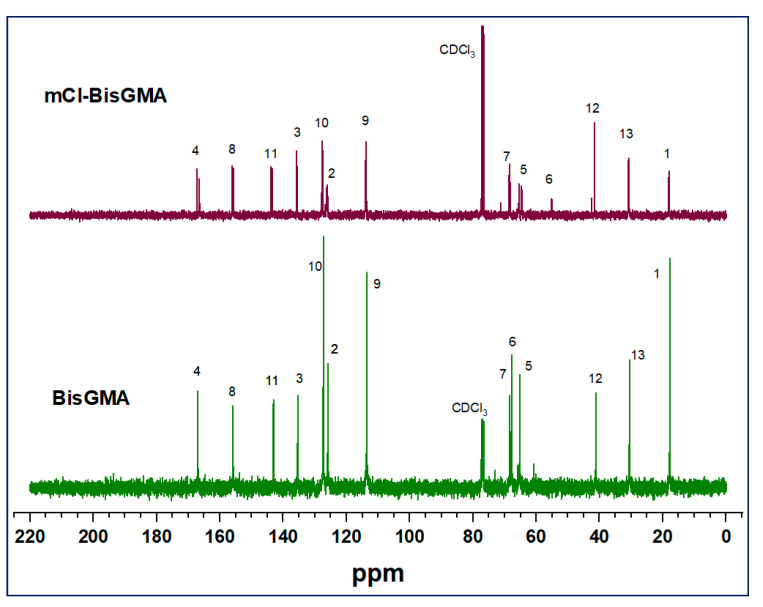
^13^C-NMR spectra of BisGMA and mCl-BisGMA in CDCl_3_. Peaks numbered in positions similar to those illustrated in ^1^H-NMR spectra.

The chemical structure of mCl-BisGMA was also analyzed using ^1^H and ^13^C-NMR spectroscopy (Figure 3 and Figure 4, respectively). As can be seen, the ^1^H and ^13^C-NMR spectral profiles of BisGMA and mCl-BisGMA are similar; however, the peaks representing the substitution center and its adjacent positions revealed a slight difference. Moreover, spectral integrations indicate the exact number of protons in both molecules. The effect of the transformation of OH-BisGMA into Cl-BisGMA analog on the NMR chemical shift has been discussed elsewhere [12]. The chemical shift of alcoholic-OH protons generally varied (0.5–5.0 ppm). This is due to the surrounding factors, including temperature, type of solvents, and concentration. Further, it is vulnerable to hydrogen bonding and, to a relatively less extent, to proton exchange [5,14,22]. Here, the singlet broad peaks (peak no. 14) at 3.9 and 3.1 ppm in the ^1^H-NMR spectra of BisGMA and mCl-BisGMA, respectively, can be attributed to the –OH protons [23,24]. Even though the intensity of the –OH proton does not always reflect the correct number of protons indicated by the integration process, the intensity of the –OH group in the spectrum of mCl-BisGMA revealed one proton (1H, H-14) compared with that in the BisGMA spectrum (2H, H-14). Similarly, the major change in the ^13^C-NMR spectrum profile of mCl-BisGMA was around the substitution center (C-5, C-6, C-7). The carbon position of the substitution center (C-6) was shifted from 67.82 ppm in BisGMA to 64.66 ppm in mCl-BisGMA, whereas relatively low chemical shifts of C-5 and C-7 from 65.22 and 68.38 to 65.49 and 68.51, respectively, was observed. All the other carbons were almost unaffected after substitution, indicating the integrity of the synthesized mCl-BisGMA. In both ^1^H and ^13^C-NMR spectra of BisGMA, some additional signals with low intensities could also be detected, particularly around the alcoholic center (positions 5, 6, and 7 in the range of 5.6–3.6) and at the low frequency range as well. However, as they are traced in the spectra of mCl-BisGMA as well, which is purified by column chromatography, these negligible peaks suggest the presence of contaminate species in the commercial BisGMA including iso-BisGMA monomer [24].

#### 3.1.3. Mass Spectrum Analysis

**Figure 5:**
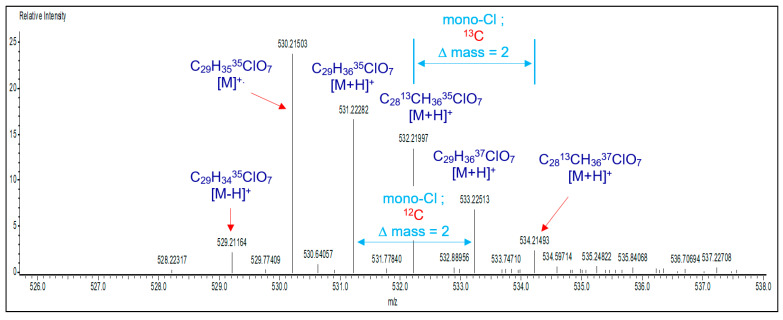
Direct analysis of mCl-BisGMA monomer in real-time mass spectroscopy (DART-MS).

To further confirm the chemical structure of mCl-BisGMA, the mass spectrum was also recorded. As shown in Figure 5, a fragment with m/z of 530.21509 was assigned to the molecular ion (C_29_H_35_^35^ClO_7_) [M]^+^. Moreover, peaks at m/z of 531.22282 and 533.22513 corresponding to (C_29_H_36_^35^ClO_7_) [M+H]^+^ and (C_29_H_36_^37^ClO_7_) [(M+H)+2]^+^, respectively, with a relative intensity of approximately 3:1 and ∆(m/z) = 2 (similarly, peaks at m/z of 532.21997 and 534.21493 for fragments with 13-carbon isotopes), characterize the presence of a single chlorine atom in the molecule [22]. This confirms the success of the one-mole –OH substitution reaction. Furthermore, peaks for [M+H]^+^ containing different isotopes, including ^13^C, ^37^Cl, or both, were observed and assigned accordingly in the spectrum.

### 3.2. Rheological Properties

#### 3.2.1. Viscosity of mCl-BisGMA

**Figure 6:**
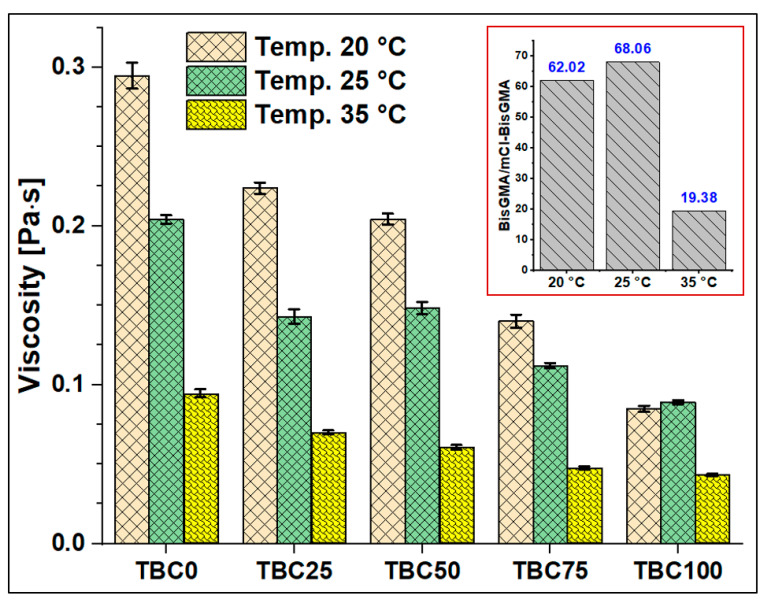
Viscosity of TBC0–TBC100 at 20, 25, and 35 °C. The inset shows an illustration of the BisGMA-to-mCl-BisGMA viscosity ratio at the three tested temperatures.

**Figure 7:**
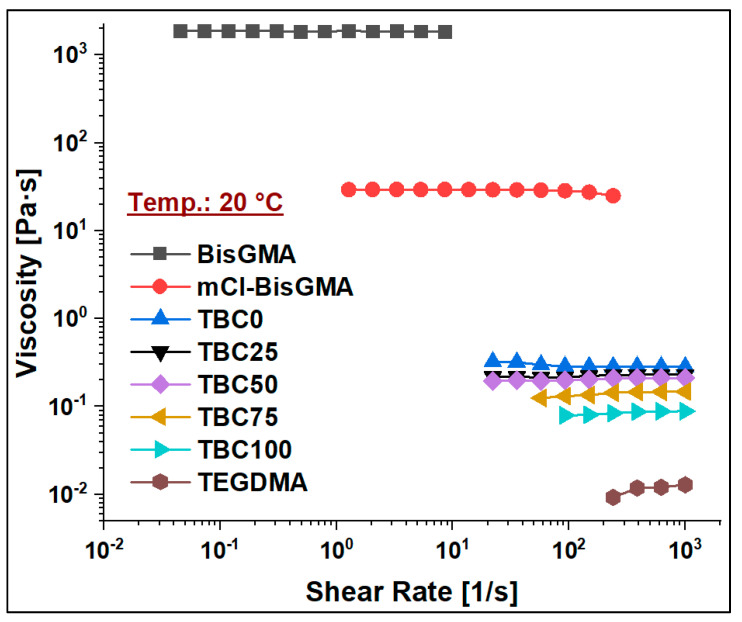
Viscosity curves of BisGMA, mCl-BisGMA, and resin mixtures TBC0–TBC100 at 20 °C.

**Figure 8:**
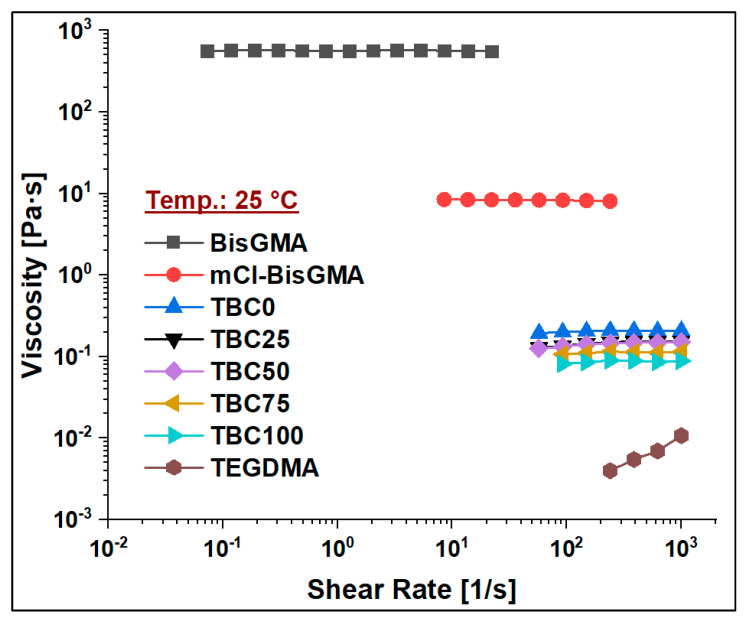
Viscosity curves of BisGMA, mCl-BisGMA, TEGDMA, and resin mixtures TBC0–TBC100 at 25 °C.

**Figure 9:**
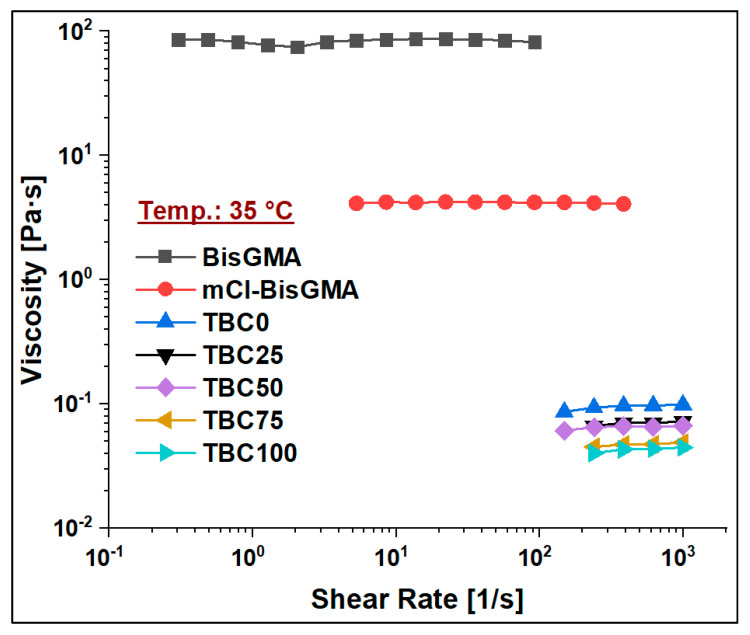
Viscosity curves of BisGMA, mCl-BisGMA, TEGDMA, and resin mixtures TBC0–TBC100 at 35 °C.

The viscosity of mCl-BisGMA was determined under the steady shear rate of a rotational rheometry mode over the range of 0.01–1000 1/s, and it was subsequently compared with that of BisGMA and TEGDMA monomers. The results of four measurements (n = 4) were averaged as given in Table 2 and Figure 6, Figure 7, Figure 8 and Figure 9. As can be seen, the viscosity was greatly enhanced after modification, e.g., from 566.1 (Pa∙s) of BisGMA to 8.3 (Pa∙s) of mCl-BisGMA at 25 °C; a similar trend in viscosity reduction at other temperatures was also observed. Reductions of approximately 62, 68, and 19 times were obtained at 20, 25, and 35 °C, respectively, Figure 6 (inset). These reductions in the viscosity of mCl-BisGMA were due to hydrogen bonding, and the reduction in mCl-BisGMA is expected to be half that in the parent monomer (BisGMA). Compared with the viscosity of dCl-BisGMA (7.22 Pa·s, measured at 22.1 °C) [12], mCl-BisGMA revealed viscosities of 29.2, 8.3, and 4.3 Pa·s at the tested temperatures of 20, 25, and 35 °C, respectively. This also indicates the great effect of the replacement of even one –OH group by Cl atom. On the molecular level, the intermolecular interaction dominantly driven by H-bonding is possibly disturbed to a degree that is comparable with the complete substitution of the two OH groups in BisGMA. Moreover, due to the loss of the pseudo-symmetry of the monomer, a substitution of one OH may also disrupt the alignment of the molecules in the bulky materials (Figure 1). The viscosity of the monomers, including BisGMA, mCl-BisGMA, and TEGDMA, reduced when temperature increased. Heating of resins promoted monomers’ thermal energy, kinetically leading to their mobility enhancement, and thus, viscosity is reduced [25,26]. Due to the considerably low viscosity of TEGDMA at high temperature, its viscosity at 35 °C was out of the device limit, and therefore, it was not reported (Figure 9 and Table 2).

#### 3.2.2. Viscosity of Resin Blends

To assess the viscosity values of resin matrices in which BisGMA was replaced by its newly synthesized analog (mCl-BisGMA), a series of resin systems in which BisGMA was incrementally replaced by 25% mCl-BisGMA (i.e., 0%, 25%, 50%, 75%, and 100%) were prepared and tested for their viscosity at different temperatures (Table 2). In dental composites, TEGDMA is the one traditional diluent monomer (η ≈ 0.01 Pa·s) used to reduce the viscosity of the resin matrix, which conventionally consists of highly viscous monomers, such as BisGMA and UDMA, as the main monomers. Thus, in this work, 50% of the investigated resins were TEGDMA (Table 1). According to the data obtained, all resins, including single and mixed monomers, exhibited Newtonian behavior over the applied shear rate, as shown in Figure 7, Figure 8 and Figure 9. The viscosity of the resin containing only BisGMA and TEGDMA (TBC0) was the highest at all temperatures, whereas TBC100 was the lowest. This could have been due to the changes in the structural properties after modification—i.e., the reduced number of H-bonds in the modified monomer (mCl-BisGMA), and the loss of the apparent molecular similarity compared with BisGMA (Figure 1), which resulted in a relatively weak intermolecular interaction, and thus, viscosity was lowered.

Table 2 reveals 3.5, 2.3, and 2.2-times reductions in the averaged viscosity of TBC0 compared with those of TBC100, from 0.29–0.08, 0.20–0.09, and 0.09–0.04 (Pa·s) at 20, 25, and 35 °C, respectively. These reductions in the viscosity of the resin matrices with an increase in mCl-BisGMA quantity indicate that the effect of H-bonding became less in the bulky material, therefore, driving down the intermolecular interaction between the components. The result of this phenomenon increased as temperature increased.

#### 3.2.3. Effect of Temperature on Resin Viscosity

Temperature is an important factor influencing the viscosities of the materials. Therefore, the viscosity of the materials under investigation, including monomers and their blends, was measured at 20, 25, and 35 °C, which may represent the ordinary, room, and body temperatures, respectively, around which material practicing are manipulated and shaped. The obtained data revealed almost a linear decrease in the viscosity values of both the monomers (BisGMA, mCl-BisGMA, and TEGDMA) and their blends (TBC0–TBC100) with temperature increase from 20 °C to 35 °C (Figure 6). This indicates that as temperature increases, the kinetic energy of the molecules increases. As kinetic energy is directly proportional to temperature, heating could reduce the forces of intermolecular interaction. Indeed, for molecules to move in a liquid, a minimum energy equal to the activated process of escape of molecules from its neighbors is required. However, the viscosity is inversely proportional to the mobility and usually obey the Arrhenius equation at least over small temperature ranges [27]. As kinetic energy increases, molecules move relatively fast, leading to a competition between weakening cohesive forces due to temperature increase and molecular interchange increase developed by the movement events of the molecules at relatively high temperatures. For high molecular weight materials, viscosity could be considerably affected by intermolecular forces represented by H-bonding, van der Waals, and dipolar interactions. Temperature is substantially a dominant factor affecting the viscosity of liquids. Consequently, in H-bonded liquids, the temperature increase causes weakening of H-bonds, and further dissociation of the intermolecular connected molecules, resulting in enhanced viscosity for the material [28].

As seen in Figure 1, BisGMA and mCl-BisGMA have relatively high molecular weights of 512.6 and 531.0 g/mol and apparent similar molecular geometries; however, mCl-BisGMA has a slightly higher molecular weight and less apparent symmetry than BisGMA. These properties may explain the significant decline in the viscosity of neat mCl-BisGMA compared with its precursor BisGMA at all temperatures. Although mCl-BisGMA molecular weight is relatively high, H-bonding, caused by –OH groups, was reduced by half. Additionally, mCl-BisGMA molecular geometry became less identical, leading to relatively low organized H-bonding and less molecular interactions. This argument could be supported by the data of TBC0–TBC100 in which the viscosity decreased with the increase in the amount of mCl-BisGMA in the resin mixtures.

Table 3 summarizes the percentage of the viscosity change with temperature, indicating the extent to which the viscosity can be affected by temperature change and providing insight into the possible working temperature. For example, when the temperature increased from 20 to 25 °C, mCl-BisGMA exhibited the highest viscosity change of 71.6%. Furthermore, the viscosity change was high in the monomers: BisGMA, 68.8%; mCl-BisGMA, 71.6%; and TEGDMA, 46.1%, which further increased as mCl-BisGMA quantity increased in the mixtures TBC0–TBC100 (30.7–3.3%); TBC100 had the lowest value.

The possible advantages of mCl-BisGMA over the other known BisGMA analogs, such as UDMA and BisEMA, are its chemical structure, molecular weight, length, and geometry, which are similar to those of BisGMA. These may conserve several BisGMA advantages when used with or in place of it. For instance, UDMA has a smaller molecular weight than BisGMA; thus, it is more flexible and less viscous; however, the core structure of most used UDMAs are varied; thus, its denomination with “urethane” in commercial products is misleading [29]. Conversely, BisEMA has a BisGMA core moiety, but its long ethylene glycol spacers cost composites some of its desirable properties, i.e., a decrease in the mechanical properties and an increase in water sorption, and subsequently, the loss of the restorative property required for its longevity [30,31]. Additionally, numerous monomers were synthesized for application as an alternative of these common monomers or to be incorporated within the traditional applicable mixtures [12,14,15,16,17].

The viscosity of the matrix generally limits the amount of the fillers used. Both the base resin and fillers are the main influencers on the properties of the dental composite and have to be controlled accordingly. To control the viscosity and improve handling characteristics of dental composites, TEGDMA, BisEMA, and some other low viscosity monomers must be added. Although the use of these diluents allow incorporation of more fillers into the matrix and increase the DC, some other adverse effects were reported, including the undesirable increase in the polymerization shrinkage and water uptake [16,32]. Even though BisGMA is commonly used as a main base for restorative composites, its huge viscosity is the major problem. However, among the various methods used for decreasing the viscosity of resins [32], lowering monomer viscosity is the appreciable one. To avoid the use of diluents, researchers have been directed toward developing low viscosity and more hydrophobic analogs of BisGMA. These properties of the base matrix allow the addition of adequate quantity of fillers (necessary for better mechanical properties) and minimizing water sorption (which induce weakening of restorative materials), respectively. For comparison purpose, the viscosity of some BisGMA derivatives reported in literature are given in Table 4. As could be seen, mCl-BisGMA has a comparable low viscosity value among the listed analogs. However, the viscosity values depend on the temperature and sometimes other measurement conditions including purity of the monomer, sampling, instrument, etc. The degree to which the viscosity of BisGMA derivative is reduced depends on many factors including the substituent type, size, and hydrophilicity-hydrophobicity characters. For instance, despite only one OH group in BisGMA was substituted, the viscosity at 25 °C of mCl-BisGMA was decreased to 8.3 (Pa·s) compared with benzoyl-, valeryl-, and methoxy-substituted BisGMA (2.7, 1.6, and 3.7 (Pa·s)), respectively, measured at the same temperature, in which the two OH groups were replaced. [14,15]. However, due to their molecular flexibility, more hydrophilic monomers commonly associated with high water solubility which is further affect the longevity of the restoratives. It could also be seen that, the viscosity of mCl-BisGMA was enhanced compared to the fluoride-containing derivatives (CF_3_-BisGM and Perfluorobutryl-BisGMA (perFB-BisGMA)) [5,33] (Table 4). Moreover, compared with dCl-BisGMA (7.2 (Pa·s) at 22.1 °C) [12], the obtained viscosity of mCl-BisGMA was satisfactory, making it one potential monomer for resin composites.

## 4. Conclusions

To overcome the drawbacks correlated with the high viscosity of BisGMA driven by H-bonding due to the presence of OH groups, the monochlorinated BisGMA analog (mCl-BisGMA) was targeted as one possible solution. This mCl-BisGMA was successfully synthesized by the Appel reaction procedure under controlled conditions for the substitution of only one OH group by one Cl atom. The pristine monomer structure was confirmed by FTIR, ^1^H and ^13^C-NMR, and MS. A considerable reduction in mCl-BisGMA viscosity was obtained compared with BisGMA viscosity (from 566.1 to 8.3 Pa·s, which is more than 68 times lower at 25 °C), and the value is comparable with the reported value for dichloro-BisGMA (7.2 Pa·s, at 22.1 °C). The replacement of BisGMA by mCl-BisGMA in resin blends also resulted in relatively low viscosities of resins; for example, the viscosity value of the BisGMA-free mixture (TBC100) was 0.09 (Pa·s), whereas that of the mCl-BisGMA-free mixture (TBC0) was 0.20 (Pa·s) at 25 °C. The low viscosity of mCl-BisGMA makes it one promising choice for the preparation of resin matrices, and its structural analogy to BisGMA may encourage further investigations to fully understand its properties as one potential dental monomer.

## Figures and Tables

**Table 1:** Compositions of the experimental resins.

Resin System	Monomer Mixture (100%)		
	TEGDMA	BisGMA	mCl-BisGMA
TEGDMA	100	0	0
BisGMA	0	100	0
mCl-BisGMA	0	0	100
TBC0	50	50	0
TBC25	50	37.5	12.5
TBC50	50	25	25
TBC75	50	12.5	37.5
TBC100	50	0	50

TEGDMA, triethylene glycol dimethacrylate; BisGMA, bisphenol A-glycidyl methacrylate; mCl-BisGMA, monochloro-BisGMA; TBC0–TBC100, resin mixtures.

**Table 2:** Viscosity of BisGMA, mCl-BisGMA, TEGDMA, and resin mixtures TBC0–TBC100 at different temperatures (Temp.); means and standard deviations (SD) of the values are indicated; n = 4.

Resin	Viscosity [Pa·s]; n = 4					
	Temp. 20 °C (V_20_)		Temp. 25 °C (V_25_)		Temp. 35 °C (V_35_)	
	Mean	SD	Mean	SD	Mean	SD
BisGMA	1813.9	24.226	566.06	6.8142	82.499	5.9326
mCl-BisGMA	29.245	0.6393	8.3165	0.1373	4.2559	0.0586
TBC0	0.2947	0.0163	0.2042	0.0054	0.0947	0.0050
TBC25	0.2238	0.0071	0.1428	0.0093	0.0700	0.0025
TBC50	0.2043	0.0071	0.1483	0.0074	0.0605	0.0030
TBC75	0.1400	0.0083	0.1119	0.0034	0.0475	0.0021
TBC100	0.0848	0.0040	0.0890	0.0025	0.0430	0.0017
TEGDMA	0.0115	0.0015	0.0062	0.0020	-	-

**Table 3:** Percentage of viscosity change with temperature.

Resin	Viscosity Change (%)		
	[(V_20_–V_25_)/V_20_)] × 100	[(V_20_–V_35_)/V_20_)] × 100	[(V_25_–V_35_)/V_25_)] × 100
BisGMA	68.8	95.5	85.4
mCl-BisGMA	71.6	85.4	48.8
TBC0	30.7	67.9	53.6
TBC25	36.2	68.7	51.0
TBC50	27.4	70.4	59.2
TBC75	20.1	66.1	57.6
TBC100	3.3	49.3	51.7
TEGDMA	46.1	-	-

**Table 4:** Comparison of the viscosity of various BisGMA derivatives.

Monomer	OH Substituent	Viscosity [Pa·s]	Temperature (°C)	Ref.
BisGMA	-	909.9	22.1	[12]
Dichloro-BisGMA	2Cl	7.2	22.1	[12]
CF_3_-BisGMA	2CF_3_	39.1	22	[33]
CH_3_-BisGMA	2CH_3_	8.4	22	[33]
Benzoyl-BisGMA	2(–OC=OC_6_H_5_)	2.7	25	[14]
Valeryl-BisGMA	2(–OC=O(CH_2_)_3_CH_3_)	1.6	25	[14]
Methoxy-BisGMA	2(–OCH_3_)	3.7	25	[15]
perFB-BisGMA	2(–OC=O(CF_2_)_2_CF_3_	54	23	[5]
Monochloro-BisGMA	Cl	29.2, 8.3, 4.3	20, 25, 35	This work

## Data Availability

The authors confirm that the data supporting the findings of this study are available within the article.
